# Cross-language phonological activation in bilingual visual word recognition: A meta-analysis

**DOI:** 10.3758/s13423-025-02692-8

**Published:** 2025-04-11

**Authors:** Nan Zhang, Zhiyi Wu, Min Wang

**Affiliations:** 1 Department of Human Development and Quantitative Methodology, University of Maryland, College Park, MD 21072 USA; 2 Graduate Program of Second Language Acquisition, School of Languages, Literatures, and Cultures, University of Maryland, College Park, MD USA

**Keywords:** Bilingual word processing, Phonological priming, Meta-analysis

## Abstract

Numerous studies have investigated whether phonological activation in the bilingual lexicon is selective or non-selective, using the classic masked priming paradigm that manipulates the phonological relatedness between primes and targets across two languages. The priming effects, however, are mixed: some studies reported reduced reaction times due to the homophone primes, while others observed non-significant priming. In this meta-analysis, we sought to systematically examine whether there is indeed cross-language phonological priming and to identify the factors that may moderate its magnitude. Analyzing 75 effects from 23 articles, we observed a significant, facilitative phonological priming effect (standardized mean difference Hedge’s *g* = 0.45, *SE* = 0.07, *p* <.0001, 95% CI = [0.32, 0.58]), hence supporting the hypothesis of non-selective activation. The moderators examined included priming direction (L1-to-L2 vs. L2-to-L1), task type (lexical decision vs. word naming), script distance (within- vs. cross-script), stimulus-onset-asynchrony (SOA), inter-stimulus interval (ISI), number of participants, as well as number of items per condition. Results revealed a significant effect of task type in cross-script studies. Specifically, the word-naming task produces a smaller priming effect than the lexical decision task. Moreover, the priming effect increases as the number of items in a condition increases. These results collectively suggested that phonological activation in the bilingual lexicon is nonselective, and the effect size is dependent upon task demands and statistical power, essential to a dual-language setting and in cross-language studies.

## Introduction

To understand bilingual language acquisition and processing, it is crucial to study how the two languages are represented and organized in the mental lexicon. One important question in this area is whether the two languages are stored integratively or separately, and a related question is whether bilinguals automatically activate the lexicons in both languages simultaneously (Brysbaert, 2003; Dijkstra & Kroll, 2005; van Heuven & Dijkstra, 2023). In the mental lexicon, phonological representation is one of the primary determinants (van Heuven & Dijkstra, 2023). Clarifying the organization of phonological information in two languages improves our understanding of bilingual lexical representation and processing. In the line of research on visual word recognition, studies have shown that phonology plays an important role in mediating the path from orthography to semantics (for a review, see Frost, 1998; Meade, 2020). This mediating role of phonology has been supported in a myriad of monolingual empirical studies (for a review, see Rastle & Brysbaert, 2006) and computational models (e.g., Dual Route Cascaded model, Coltheart et al., 2001; for a review, see Norris, 2013). Cross-language phonological activation in bilingual visual word recognition, however, was largely overlooked until the publication of Gollan et al. (1997), Dijkstra et al. (1999), and Brysbaert et al. (1999). Since then, automatic cross-language phonological activation has been tested in both non-priming word recognition tasks (e.g., Carrasco-Ortiz et al., 2021; Comesaña et al., 2015; Dijkstra et al., 2010; Friesen et al., 2014; Lemhöfer & Dijkstra, 2004; Peleg et al., 2020; Sáchez-Casas et al., 1992; Schwartz et al., 2007) and priming tasks, especially the masked priming tasks (e.g., Ando et al., 2014; Brysbaert et al., 1999; Dimitropoulou et al., 2011; Duyck et al., 2004; Kim & Davis, 2003; Lim & Christianson, 2023; Nakayama et al., 2012; Wijnendaele & Brysbaert, 2002; Zhou et al., 2010). The current meta-analysis focused on evidence obtained from masked priming tasks where the target is presented following a mask (i.e., a string of symbols) and a brief presentation of the prime. In the masked priming paradigm, the primes are automatically processed, which prevents strategic responses, thus providing compelling evidence for non-selective activation. A vast number of masked priming studies adhering to this standard design have been carried out to study lexical representation and processing during visual word recognition in the past decades. In our meta-analysis, we investigated the robustness of cross-language phonological priming under various conditions and identified factors that may moderate the strength of this effect.

## Theoretical models of bilingual lexical activation

Various models have explained the representation and processing of two languages among bilingual speakers (e.g., Sense Model, Finkbeiner et al., 2004; Revised Hierarchical Model, Kroll & Stewart, 1994; Kroll et al., 2010). Among them, the Bilingual Interactive Activation Plus Model (BIA +; Dijkstra & Heuven, 2002), a verbal model, and the Multilink Model (Dijkstra et al., 2019), a computational model, are two leading models contributing to our understanding of bilingual phonological activation during visual word recognition.

The BIA + model proposes an integrated lexicon and parallel activation, where the orthographic and phonological representations (collectively, lexical representations) of two languages are stored in a unified mental lexicon at both lexical and sublexical levels. Additionally, semantic information is integrated across the two languages, and a language code decides the language membership of the visual input. The orthographic, phonological, and semantic representations, plus the language code, jointly form the word identification system. In this model, a specific stimulus non-selectively activates all related words in both languages that share lexical and semantic representations with the stimulus (e.g., when the French word *fille* is given, the English word *fee* will be activated simultaneously; Friesen et al., 2020). Context and task requirements can mediate lexical processing and behavioral responses by assigning different weights to the different types of lexical representation. A task schema incorporates a series of cognitive operations or actions that lead to a pre-specified goal, for example, correctly performing a word-naming task (Lam & Dijkstra, 2010). In this phonologically demanding task, the activation of phonological representation would be more necessary.

Recently, the localist-connectionist Multilink model has been introduced (Dijkstra et al., 2019). Similar to the structure of the BIA + model, the Multilink model incorporates a symbolic lexical network that contains layers of orthography, phonology, semantics, and language membership, as well as task/decision process. Going beyond the verbal explanation in BIA + model, it simulates the recognition of three- to eight-letter words including cognates of different lengths. The current Multilink model does not include the sublexical level, instead, focuses primarily on the interactions between lexical forms and their meanings, and phonological activation linked directly to these forms. In the BIA + model or Multilink model, there exists a feedforward from phonology and orthography to language nodes. However, there is no top-down influence from language nodes to phonology and orthography. Furthermore, the Multilink model takes into account the frequency of usage, length, and cross-linguistic similarity of words in the two languages, which helps explain the effects of language proficiency and cross-language differences in bilingual lexical processing. Some of the critical assumptions made in the Multilink model are as follows: first, there are no lateral inhibitory effects between words within or between languages; second, language access is non-selective, and word neighbors are activated parallelly. In other words, upon receiving a visual input, the word itself and its interlingual homophone will be automatically activated without inhibition. Furthermore, the Multilink model emphasizes that the activation of competitors directly depends on the orthographic overlap between the input word and stored lexical representation. The greater orthographic overlap, the stronger activation of competitors.

## Cross-language masked phonological priming

In the studies that used the masked priming paradigm to investigate cross-language phonological activation in visual word recognition, researchers manipulated the phonological relatedness between primes and targets (related vs. unrelated) and compared the reaction time (RT) and accuracy of participants’ responses to the targets in the two conditions. The faster and more accurate responses to the targets preceded by phonologically related primes indicate that phonological representation is shared across languages and automatically, non-selectively activated in both languages. To avoid strategic and biased processing of the primes, researchers use a mask to prevent participants from consciously recognizing the prime, ensuring subliminal processing (for a review, see Forster et al., 2003).

Two types of phonologically contrasting conditions have been employed depending on whether primes and targets are semantically related. The first type involves the comparison between interlingual homophones and a set of graphemic control words, with no semantic overlap between primes and targets. The graphemic control words have no phonological overlap with the targets, but the orthographic overlap is similar between control words and targets and between homophone primes and targets (e.g., Dutch-French: Brysbaert et al., 1999; Duyck et al., 2004; Greek-Spanish: Dimitropoulou et al., 2011; Korean-English: Kim & Davis, 2003; Chinese-English: Zhou et al., 2010). For instance, in Brysbaert et al. (1999) where Dutch–French bilinguals were tested on a masked priming lexical decision task, the primes are interlingual homophones to the targets in the phonologically related condition (e.g., wie-OUI; translation: who-YES), whereas in the unrelated condition, the primes are phonologically different from the targets (e.g., jij-OUI; translation: you-YES). The primes in both the phonologically related and unrelated conditions share the same letters at the same positions with the target words (in the phonologically related example wie-OUI, the overlapping letter is i in the second position; in the control example jij-OUI, the overlapping letter is also i in the second position). A facilitative phonological priming effect was revealed by the higher accuracy in the related condition. Some other priming studies measured the facilitation of phonological primes via faster RT in the related condition (Korean-English: Kim & Davis, 2003; Chinese-English: Zhang et al., 2024; Zhou et al., 2010).

The second approach manipulates the phonological similarity of translation counterparts. Researchers either compare cognates (translation counterparts that have form overlap) with non-cognate translations while controlling for the degree of orthographic overlap between the cognate pairs (e.g., Chen et al., 2022; Gollan et al., 1997; Nakayama et al., 2012) or compare cognate pairs that are more phonologically similar with those that are less phonologically similar (e.g., Nakayama et al., 2014). For example, Gollan et al. (1997) compared participants’ response latency to a target primed by its cognate (e.g., פילטר/feelter/–FILTER) with that to a target primed by its non-cognate translation (e.g., ארמון/armon/–CASTLE). Since the two conditions were not matched on word frequency, two groups of control words were created for the cognate and non-cognate conditions respectively (e.g., for cognates: גרגיר/gargir/, meaning berry; for non-cognates: סיגלית/sigalit/, meaning violet). The translation priming effect in the cognate condition was larger than in the non-cognate condition in terms of RT, providing evidence for cross-language phonological priming.

To date, although findings have been relatively consistent regarding the facilitative role of phonological overlap in the simultaneous activation across languages (for a review, see Jiang, 2023), the magnitude of the phonological priming effect as well as its moderating factors have not been systematically examined. For example, Kim and Davis (2003) failed to observe a priming effect in a lexical decision task among Korean-English bilinguals, whereas Nakayama et al. (2012) and Ando et al. (2014) both showed a significant masked priming effect in Japanese-English bilinguals’ lexicality judgment. The discrepancy could be due to language pairs and the varied choice of masking and different priming procedures, including the type and duration of the mask, duration of the prime, and interval between the prime and the target. Taken together, there is a clear gap in the literature in clarifying the underlying factors that may impact phonological priming. In the following sections, we reviewed multiple potential factors, including those related to the stimuli (priming direction and script distance), task type, task procedure (SOA and ISA), and statistical power (number of items and participants). The extent to which these factors may moderate the cross-language phonological priming was evaluated.

## Moderators

### Stimuli

#### Priming direction

Priming direction (from L1 to L2 or from L2 to L1) is a stand-out factor that has received ample attention in cross-language activation research. The varying priming effects in different directions may point to varying strengths of links between L1 and L2 lexical representation and the varying quality of representation in the two languages. According to the Fuzzy Lexical Representation Hypothesis (Gor et al., 2021), phonological representation in L1 is stored in a higher quality and accessed more rapidly compared to that in L2, it is expected that priming would be larger in the direction of L1 to L2 than the other way around. Zhou et al. (2010) showed evidence supporting this hypothesis, with a larger priming effect from L1 to L2 (12 ms in word naming and 21 ms in lexical decision) than from L2 to L1 (8 ms in word naming and 18 ms in lexical decision). However, other studies, such as Xu et al. (2021), reported an opposite pattern, with a significant priming effect shown from L2 to L1 (14 ms) but not from L1 to L2 (9 ms) in a masked word-naming task under a similar SOA condition (43 ms). Earlier findings in a Dutch–French study (van Wijnendaele & Brysbaert, 2002) added further nuance to this pattern, as the phonological priming shown from Dutch L1 to French L2 was similar to that from L2 to L1 (7 ms vs. 6 ms). Thus, the current meta-analysis sought to systematically examine to what extent priming direction influences phonological priming.

#### Script distance

A script is defined as an expression of the visual appearance of a written language (Perfetti & Dunlap, 2012). There are different types of scripts representing different languages in the world, for example, European scripts (Roman, Cyrillic, Greek), Indian scripts (Hindi, Bengali, Tamil), Semitic scripts (Arabic, Hebrew), and Chinese/Japanese traditional scripts. When two languages used by a bilingual share the same script category, they are considered within-script. For example, English and Spanish, as well as Dutch and French, use the Roman alphabet and thus are classified as within-script. Conversely, if two languages employ different script categories, such as Hebrew (Semitic script) and English (Roman script), they are classified as cross-script. Languages employing different scripts tend to exhibit a reduced degree of orthographic overlap compared to those using the same script (Dijkstra et al., 2023; Miwa et al., 2014; Mountford, 2002).

Researchers hold different viewpoints concerning the influence of orthographic distance on cross-language phonological priming. In favor of the selective lexical activation hypothesis, Gollan et al. (1997) believed that when the scripts are different between the two languages utilized in a priming study, readers may use orthographic cues to intentionally access the specific lexicon of the prime and target. This allows for prompt access to the correct lexicon, increasing the chance of priming. In addition, a large script distance lowers the orthographic inhibition exerted on the words in the other language and enhances cross-language co-activation, thus leading to significant priming (Dimitropoulou et al., 2011; Kim & Davis, 2003). However, some other researchers support the language non-selective hypothesis (e.g., Nakayama et al., 2012) and postulate that phonological representation and activation may be suppressed due to low orthographic similarity when the two languages do not share the same script. Consequently, the cross-language phonological priming observed in same-script languages may be reduced or even absent in different-script languages.

Findings concerning cross-language phonological priming are relatively consistent in within-script studies. For instance, Brysbaert et al. (1999) used a word perceptual identification task and found a significant phonological priming effect from Dutch L1 to French L2. In this task, a 42-ms display of a prime (either a real word or a pseudoword in Dutch) was followed by a target word (presented for 28 ms or 42 ms) and a postmask (“XXXX”). Participants were instructed to type the word they perceived. The primes were either phonologically similar (e.g., wie-OUI, meaning *who*-*you* in English) or unrelated (e.g., jij-OUI, meaning *yes*-*you* in English) to the target French real words. Each prime in the unrelated condition and its corresponding prime in the similar condition had the same overlapping letter with the target (e.g., the letter *i* in the example) to minimize differences in orthographic similarity between the prime and the target. The phonological priming effect was calculated by subtracting the accuracy rate in the orthographic control condition from that in the phonologically similar condition. Results revealed a facilitative effect. This finding was replicated by van Wijnendaele and Brysbaert (2002) and Duyck et al. (2004) who examined phonological priming from French L2 to Dutch L1.

In contrast to the consistent findings in within-script studies, evidence regarding phonological priming is mixed in cross-script studies. Significant phonological priming has been observed in various patterns: only from L1 to L2 (Hebrew-English: Gollan et al., 1997; Chinese-English: Zhang et al., 2024), only from L2 to L1 (Chinese-English: Xu et al., 2021), or in both directions (Chinese-English: Zhou et al., 2010). For instance, Zhou et al. (2010) showed significant bidirectional phonological priming among Chinese-English bilinguals in a word-naming task, whereas Xu et al. (2021) found no priming effect from Chinese L1 to English L2 using the same task. Comparing within- and cross-script studies, the orthographic characteristics of a language may play a role. However, the magnitude of the effect due to varying orthographic overlap has rarely been directly compared, except by Dimitropoulou et al. (2011), who observed a larger cross-language phonological priming effect among Greek-Spanish bilinguals when the orthographic overlap between the prime and the target was higher compared to when the orthographic overlap was lower. A systematic, quantitative comparison of phonological priming between within-script and cross-script studies can help understand the extent to which the orthographic distance mediates cross-language phonological activation.

### Task type

According to the BIA + and the Multilink models, task type in visual word recognition studies can modulate lexical processing by weighing the phonological, orthographic, and semantic codes differently based on specific task requirements. These tasks include deciding the lexicality of a word (lexical decision) or the language of a word (language decision), reading a word aloud (word naming), categorizing words based on semantics (semantic categorization), or writing down words after a very brief presentation (perceptual identification). Empirical data from various tasks yielded different conclusions concerning whether automatic cross-language activation is present. Among these tasks, word naming and lexical decision have been the most frequently used word recognition tasks (Ferrand et al., 2011). These two paradigms are used to study the extent to which phonology is involved in visual word recognition (Katz et al., 2012) in both monolingual studies (Coltheart, 1985; Gao et al., 2016; Kinoshita & Norris, 2012; Zhang et al., 2018) and in bilingual studies (e.g., De Groot, 2011; De Groot et al., 2002; Haigh & Jared, 2007; Jared & Kroll, 2011; Jiang & Pae, 2020; Peleg et al., 2020; Tiffin-Richards, 2024; Xu et al., 2021). The contrast between these two most common tasks provides a better understanding of the nature of form activation in bilingual lexical processing. The difference in task demands between these two tasks stems from whether phonological information is necessary to complete the tasks. In a lexical decision task, where participants decide whether the target is a word, they could utilize phonological, orthographic, or semantic information to complete the task, making access to phonological representation optional. In contrast, a word-naming task requires participants to pronounce the target word, necessitating access to its precise phonological representation. Thus, comparing these two tasks could reveal whether phonological priming is modulated by task requirements related to phonological information. In addition to lexical decision and word naming, the perceptual identification task was used in the early phase of this line of research (e.g., Brysbaert et al., 1999; Brysbaert & Van Wijnendaele, 2003; Duyck et al., 2004; Van Wijnendaele & Brysbaert, 2002). The current meta-analysis compared phonological priming effects across these three tasks: lexical decision, word naming, and perceptual identification.

### Task procedure

#### Stimulus onset asynchrony (SOA) and inter-stimulus interval (ISI)

The SOA refers to the time interval between the onsets of the prime and the target, consisting of the prime duration and the ISI (i.e., the interval between the offset of prime and the onset of target). The length of SOA determines the time allowed for participants to perceive and process the prime words in priming studies, which in turn affects the prime’s visibility and the priming effect size. The insertion of an ISI is known to increase the visibility of the prime (Forster et al., 2003). The relationship between SOA and the magnitude of the priming effect is complex. Firstly, a short SOA reduces the prime’s visibility, but an excessively brief SOA could lead to partial or null phonological priming. For example, Brysbaert et al. (1999) found a facilitative effect of homophone primes with an SOA of 42 ms but not when it was 27 ms. Secondly, within a specific range, the phonological priming effect becomes larger as the SOA increases, because a longer SOA grants participants more time to access and identify the prime. Forster (1999, 2003) revealed that the magnitude of priming effects increases linearly with the growth of SOA when the SOA is longer than 20 ms and shorter than 50 ms in repetition and orthographic priming studies, while the priming effect grows nonlinearly as the SOA exceeds that range (e.g., 60 ms). Thirdly, an extremely long SOA might decrease the phonological priming effect, since the unmatched orthography suppresses the shared phonological representations. For instance, Lukatela and Turvey (1994) observed that an English target word was primed by a homophone of its semantically associated word (e.g., frog by toad-towed) at an SOA of 57 ms, but this priming effect vanished when the SOA reached 250 ms. However, phonological priming was still shown at long SOAs with pseudoword primes (cf. Drieghe & Brysbaert, 2002).

The effects of SOA and ISI were supported by a meta-analysis on the masked non-cognate translation priming effect (Wen & van Heuven, 2017), where SOA and ISI accounted for 26.25% of the variance in translation priming effects from L1 to L2. Another meta-analysis on the same topic (Davis & Kim, 2021) showed that studies using SOAs longer than 60 ms had significantly larger translation priming effects than those shorter than 60 ms. However, no previous systematic review has examined how cross-language phonological priming is moderated by SOA and ISI. In the current meta-analysis, we included them as two separate moderators in the regression analysis to study their individual influence on phonological priming.

### Statistical power

The number of participants and the number of stimuli determine the statistical power of an analysis. For instance, in a classic study on cross-script phonological priming (Kim & Davis, 2003), researchers failed to show homophone priming from Korean to English, possibly due to the small number of participants (*N* = 18). Adopting a similar set of materials, Lim and Christianson (2023) increased the sample size of participants to 60 and identified a significant phonological priming effect. These different findings suggest that the number of participants may play an important role in the significance of statistical tests. We sought to systematically examine whether this factor indeed affects phonological priming across a wide range of studies.

## Previous meta-analyses on phonological priming

Previous meta-analyses have examined semantic activation in monolingual studies (van den Bussche et al., 2009) and non-selective activation of semantics (Davis & Kim, 2021; Lauro & Schwartz, 2017; Wen & van Heuven, 2017) in bilingual studies. However, cross-language phonological activation has only been synthesized in narrative reviews (e.g., Brysbaert, 2003; Desmet & Duyck, 2007; Jiang, 2018, 2023). The first narrative review on automatic cross-language phonological activation in visual word recognition (Brysbaert, 2003) examined the evidence for three hypotheses based on two masked perceptual identification studies (in total three experiments; Brysbaert et al., 1999; van Wijnendaele & Brysbaert, 2002). These hypotheses state that (1) phonology plays a similarly crucial role in mediating L2 visual word recognition as it does in L1, (2) L1 words can prime L2 homophones, and (3) L2 words can prime L1 homophones. This review provides a detailed historical perspective on research concerning phonological processing in both L1 and L2. While the scope of studies included is limited to Dutch and French, reflecting the available research at the time, it meticulously compiled and analyzed the findings from the available literature to offer valuable insights into the field. After Brysbaert’s (2003) work, there were more narrative reviews to synthesize subsequent studies on cross-language phonological activation (e.g., Desmet & Duyck, 2007; Jiang, 2018, 2023). However, these reviews covered a wide aspect of bilingual processing, such as orthography, phonology, and morphology, with phonological activation being a small section. In addition, these reviews did not conduct a systematic search or perform a quantitative synthesis of previous research on phonological activation.

Albeit the lack of meta-analyses specifically focused on cross-language phonological activation, the previous meta-analytic reviews of monolingual phonological processing, particularly those focusing on masked priming tasks, can provide us with valuable insights. One such meta-analysis investigated the robustness of masked phonological priming in monolingual English speakers (Rastle & Brysbaert, 2006). This review systematically scrutinized the literature on masked priming and categorized 20 eligible studies (107 effect sizes) into five groups based on task and procedure: forward-masked perceptual identification, backward-masked perceptual identification, forward-masked reading aloud, forward-masked visual lexical decision, and text reading tasks. Within each group, the researchers found an overall statistically significant homophone priming effect across the studies. However, the effect sizes across the five groups of studies were not compared with each other. Additionally, the potential moderators that may help explain the heterogeneity in priming were not examined, such as the priming paradigm (i.e., forward- vs. backward-masked), task demand (i.e., perceptual identification, reading aloud, vs. visual lexical decision), or context (i.e., isolated word vs. text reading). Furthermore, this meta-analysis solely examined monolinguals whose English was L1, making it difficult to generalize the findings to other languages and bilingual phonological activation.

More recent meta-analyses have examined cross-language activation with a focus on masked semantic priming (van den Bussche et al., 2009) and masked non-cognate translation priming (Davis & Kim, 2021; Wen & van Heuven, 2017). These studies compared the pooled priming effects against zero and investigated potential moderators via meta-regression. Wen and van Heuven (2017) analyzed non-cognate translation priming effects in 64 masked priming lexical decision experiments from 24 studies and showed a significant non-cognate translation priming effect both from L1 to L2 (standardized mean difference *d* = 0.86) and from L2 to L1 (*d* = 0.31). Seven moderators were examined, including the number of participants, the number of items per cell, the prime duration (in ms), the ISI (in ms), the SOA (in ms), the overall response speed (average reaction time across experimental and control conditions), and the script type (same- vs. different-script). Both ISI and SOA were significant predictors for L1-to-L2 priming, whereas the number of items per cell was a significant predictor for L2-to-L1 priming. Davis and Kim (2021) synthesized masked priming studies (76 effects) on non-cognate translation activation in two priming directions. A significant priming effect was found for both directions, with a significantly larger effect from L1 to L2 than from L2 to L1 (L1 to L2: *d* = 0.79, L2 to L1: *d* = 0.29). The priming effects were then regressed on three moderators: script type (same- vs. different-script), SOA (≤ 60 ms vs. > 60 ms), and reading experience (high vs. mid-low). The three moderators combined significantly predicted the effect sizes from L1 to L2, but not from L2 to L1. Findings from the above meta-analytical reviews of semantic and translation priming provide valuable insights into the potential moderators of cross-language phonological activation in the current study.

## The current meta-analysis

The following questions guided our meta-analysis: (1) Is there an overall significant masked cross-language phonological priming effect in bilingual visual word recognition? In other words, are RTs^1^ in the phonologically related condition significantly reduced compared to those in the phonologically unrelated condition? (2) Is the priming effect moderated by characteristics of stimuli (priming direction and script distance), task type, task procedure (SOA and ISI), and statistical power (numbers of participants and items in each condition)? We also investigated if there are significant interactions among priming direction, script distance, and task type.

Based on previous literature, we hypothesized an overall significant masked phonological priming in RT. Second, we predicted that the priming effect would be influenced by the following moderators: stimuli, task type, task procedure, and statistical power. For example, the priming effect may be larger from L1 to L2 than that from L2 to L1. Furthermore, we hypothesized that the effects of priming direction and task type might be different between within-script studies and cross-script studies. In cross-script studies, phonological priming from L1 to L2 may be larger than that from L2 to L1, while this asymmetry may not be present in within-script studies. This hypothesized discrepancy is based on the potential suppression of phonological activation due to low orthographic similarity in cross-script languages. The extent of this suppression might vary depending on how much L1 and L2 differ in their orthography-phonology linkage, that is, the possibility and strength of activation from orthography to phonology. When L1 and L2 share the script, the orthographic similarity between the prime and the target is relatively high, leading to a comparable strength of the orthography-phonology links in both languages. When L1 and L2 employ different scripts, the lower prime-target orthographic similarity may lead to a differential strength of the link between orthographic form and phonological representation in the two languages. Thus, the phonological activation from the more proficient L1 is likely to be stronger than that from the less proficient L2, resulting in an effect of priming direction.

## Methods

### Literature search and inclusion criteria

**Fig. 1:**
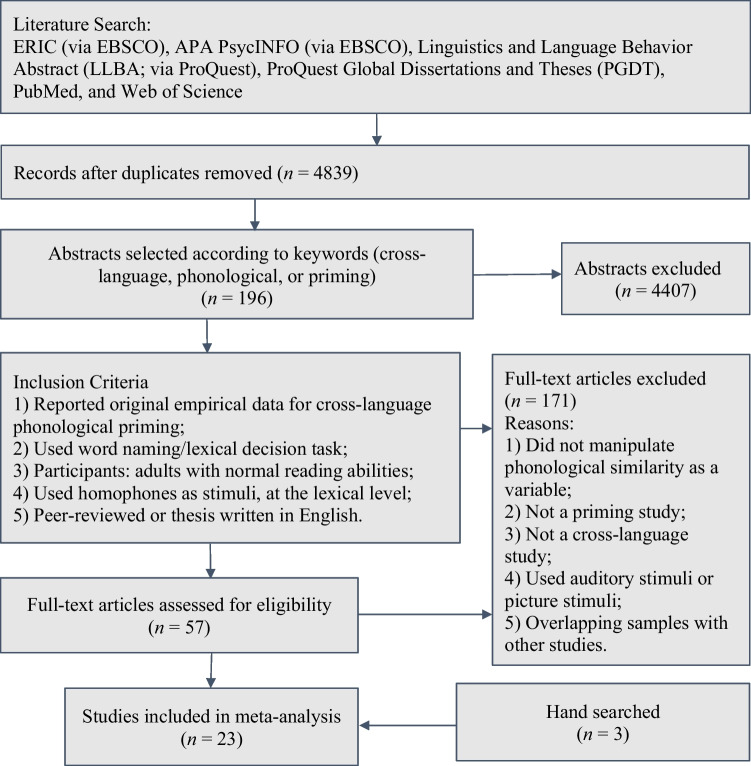
Flow diagram for the literature searching and inclusion criteria

We included 23 studies (*k*_*1*_ for the number of studies hereinafter) that yielded 75 effects (*k*_*2*_ for the number of effects hereinafter) in the meta-analysis. We searched and screened the literature following the suggestion by Preferred Reporting Items for Systematic Reviews and Meta-Analyses statement (PRISMA 2020 version; Page et al., 2021). A flow chart illustrating the procedure of the literature search and screening is presented in Fig. 1.

#### Search strategy

A comprehensive search was conducted in six online databases including ERIC (via EBSCO), APA PsycINFO (via EBSCO), Linguistics and Language Behavior Abstract (LLBA; via ProQuest), ProQuest Global Dissertations and Theses (PGDT), PubMed, and Web of Science. Our search strategy involved using the keywords bilingual OR cross-language OR"cross language"OR cross-script OR"cross script"OR second-language OR"second language"AND phonolog* OR homophone* AND priming OR prime* in the full text of all databases except for PGDT, where we searched within abstracts only. This search strategy returned 4,839 articles published between 1970 and August 2023. We used Rayyan (http://rayyan.qcri.org; Ouzzani et al., 2016) to further screen the article manually. After removing duplicates, 4,407 records were retained. Then, 57 potentially eligible articles were retained based on the full text. Using the inclusion criteria which are discussed in the following section, we retained 20 articles for coding. After this round of full-text screening, we hand-searched reference lists and tracked forward citations of the included articles to identify additional studies that may have been missed and found three more studies. In total, 23 articles (*k*_*2*_ = 75) were included. Unpublished theses and dissertations were included to address the publication bias. Among the total 75 effects, 12 effects were from unpublished research (three dissertations).

#### Eligibility criteria

The included studies are limited to peer-reviewed journal articles, manuscripts in preparation, and master’s or doctoral theses written in English and met all the following criteria:

1. Participants were bilingual adults with normal reading abilities and aged between 18 and 60 years. Studies with a focus on populations with dyslexia, hearing, or visual impairments were excluded, so were studies that involved sign languages.
2. Studies tested a masked priming effect, with primes briefly displayed and masked (less than 100 ms; see the meta-analysis by van de Bussche et al., 2009 where the same criterion was used). Two studies were excluded due to a long ISI (Singh et al., 2022, of which the ISI was 550 ms) or a long prime duration (Lee et al., 2005, in which the SOAs were 140 ms and 250 ms; see Jiang, 2023, for the justification that primes are visible under a long SOA).
3. The prime and the target were in two different languages. Studies that looked at two scripts of the same language (e.g., Rao et al., 2011, which used Roman-script-transcribed Urdu to prime Urdu) were excluded.
4. Studies manipulated the phonological similarity between primes and targets. That is, primes and targets had either full or partial phonological overlap in the phonologically related condition and little phonological overlap in the unrelated condition. For instance, among the included articles, Jouravlev et al. (2014) and Timmer et al. (2014) examined the masked onset priming effect, where primes and targets shared the onset in the phonological-related condition; Nakayama et al. (2012) examined the homophone priming effect, where primes and targets share the syllable in the phonological-related condition.
5. Eligible studies controlled for the influence of orthographic overlap, word frequency, and word length on the phonological priming effect. Since languages with the same script tend to have orthographic overlap among homophone pairs, only effects where orthographic similarity was controlled for were included in our analysis. For instance, in Brysbaert et al. (1999), the homophone pair “wie”–“OUI” has one letter “i” in common, so they added a graphemic control word “jij” that has the same letter “i” in the same position as in “wie”. In studies that compared cognates (phonologically similar) and noncognate translations (phonologically unrelated), the two types of words were matched with two groups of orthographic control words respectively. Furthermore, cognate and noncognate words as targets were matched on word frequency and length.
6. Studies used a lexical decision, a word-naming, or a perceptual identification task with written visual stimuli to investigate bilingual visual word recognition. Studies incorporating auditory stimuli or picture stimuli were excluded.
7. Studies provided either conditional means and standard deviations, or *t* or *F* statistics with the degree of freedom of numerator as 1 (e.g., Rosenthal, 1995). In cases where a study met other inclusion criteria but failed to provide necessary statistics for calculating the synthesized effect, the first author contacted the authors of that study for more information. Out of the four inquiries made, two (Li, 2021; Liu et al., 2023) supplied the necessary information for our current meta-analysis, while the other two studies (Lee-Kim et al., 2021; Voga & Grainger, 2007) were excluded due to insufficient statistical data for effect size calculation.

### Coding process

In line with previous meta-analyses on masked priming studies (e.g., Rastle & Brysbaert, 2006; Van den Bussche et al., 2009; Wen & van Heuven, 2017), we developed a coding manual that encompassed the identification number (ID) of each effect (e.g., the Chinese-English phonological priming in lexical decision in Zhou et al., 2010), moderator, and the size of priming effect (e.g., 10 ms for the first effect in Zhou et al., 2010). The first and the second authors independently carried out the coding process for all included studies and cross-checked the results, adhering to Wilson’s (2009) recommendation for systematic coding. The inter-rater reliability for all studies yielded a high level of agreement, with a 94.76% consensus between the two coders. All discrepancies were resolved through discussion until consensus was achieved. Four types of moderators related to stimuli, task type, task procedure, and statistical power were included in the coding and analysis.

#### Stimuli

The first category of the moderators, stimuli, includes *priming direction* and *script distance*. Priming direction was coded as L1-to-L2 or L2-to-L1. The script distance was coded as within-script or cross-script depending on whether the primes and targets use the same script. The languages involved in the meta-analysis are Chinese, English, French, Dutch, Hebrew, Korean, Japanese Kanji, Japanese Katakana, Russian, and Urdu. For alphabetic languages, English, French, Dutch, and Spanish use Roman script; Russian uses Cyrillic script; Hebrew uses Arabic script; Urdu uses Persian script which was derived from Arabic (Mountford, 2002). Three Asian languages, Chinese, Japanese (only its Kanji and Katakana writing systems were utilized in the included studies), and Korean, all have their unique scripts. Therefore, the script distance of the 11 language pairs included in the meta-analysis was coded as follows: within-script pairs are Dutch–English, Dutch–French, and English–French; Cross-script language pairs include Chinese–English, English–Hebrew, Greek–Spanish, Japanese Kanji–English, Japanese Katakana–English, Urdu–English, Russian–English, and Korean–English. The script type and a sample of written words of each language are provided in Table 1.

**Table 1:** Script type and word example of languages involved in the priming studies

	Script Pairs (Examples)	*k*_*2*_	Script1 Category	Script2 Category
Same-script	**Dutch–**English	2	Roman	Roman
	(**wie–**we)			
	**Dutch–**French	4	Roman	Roman
	(**wie–**oui)			
	**English–**French	6	Roman	Roman
	(**we–**oui)			
Cross-script	**Chinese–**English	29	Ideographic/Logographic	Roman
	(**维–**we)			
	**English–**Hebrew	4	Roman	Semitic
	(**we–**אָנוּ)			
	**Greek–**Spanish	2	Greek	Roman
	(**μωρό–**mora)			
	**Japanese (Kanji) –**English	2	Ideographic/Logographic	Roman
	(**木–**key)			
	**Japanese (Katakana) –**English	5	Phonographic	Roman
	**(ラッキー–**lucky)			
	**Korean–**English	12	Phonographic	Roman
	**(펜**-pen)			
	**Russian–**English	1	Cyrillic	Roman
	**(кaлaч–**claim)			
	**Urdu–**English	8	Persian, derived from Arabic	Roman
	(کاغذ**–paper**)			
Total		75	-	-

The script pairs listed here do not differentiate the two priming directions

#### Task type

The *task type* was coded as a categorical variable. Within the 23 included articles, there are three types of tasks, i.e., the lexical decision task, the word-naming task, and the perceptual identification task. Fifty effects from 16 studies used the lexical decision task, 19 effects from ten studies used the word-naming task, and six effects from four studies used the perceptual identification task.

#### Task procedure

The task procedure includes SOA and ISI, which were treated as continuous variables.

#### Statistical power

The final category of the moderators includes *the number of participants* as well as *the number of items* in the phonologically related and control conditions respectively. Only items included in the data analysis were considered.

To compute the effect size, we incorporated conditional means, standard deviations, and *t* or *F* statistics.

### Procedure

#### Effect size calculation

The outcome of interest is the difference in RT between the phonologically related and the unrelated conditions. The phonological priming effect can be obtained in two ways. The first method is to compare the RT in the phonologically related condition with that in the control condition. The calculation of 59 effect sizes followed this approach. Among these, the prime in the experimental condition in 55 effects had full phonological overlap with the target. In the other four effects (Jouravlev et al., 2014; Kim & Davis, 2003; Timmer et al., 2014), the prime and the target shared the onset in the phonologically related condition. The second method is to compare the cognate priming with the noncognate translation priming effects, applied to the remaining 16 effects. In these effects, the stimuli in the phonologically related condition were not matched for word frequency and length with those in the unrelated condition (or in high vs. low phonological similarity conditions, as in Nakayama et al., 2014). This discrepancy occurred because the examination of the phonological priming was not their primary focus. Instead, such lexical properties of the stimuli in each experimental condition were matched with those in two different control groups, respectively. For both approaches, a positive value of the coded effect size indicates a facilitatory phonological priming effect. More details for effect size calculation can be found in the following section. A positive value means a facilitatory effect. Note that for studies that did not report RT as the outcome (i.e., Brysbaert et al., 1999; Duyck et al., 2004; Haigh, 2008; van Wijnendaele & Brysbaert, 2002), the error rate (ER) was used instead.

We used the following formulas to calculate the standardized mean difference (SMD), Hedge’s *g*, which corrects the sample size bias by multiplying a correction factor *J* by Cohen’s *d*. *J* is calculated as follows:

$$J = 1-\frac{3 }{4N-9},$$

in which *N* is the number of participants in each effect. Depending on the available statistical data in each article, four equations were used to calculate Cohen’s *d*, reported below.

If means and standard deviations of the phonologically related and control conditions are reported, we calculated *d*_*z*_ as Cohen’s *d* via

$${d}_{z} =\frac{{M}_{RT1i}-{M}_{RT2i} }{{SD}_{pi}},$$

in which *d*_*z*_ is the standarzed difference score for a within-subject design, obtained from the raw difference between two conditions divided by the *SD* of difference (Cohen, 1988; Jané et al., 2024). ${M}_{RT1i}$ is the mean RT in the control condition, ${M}_{RT2i}$ is the mean RT in the phonologically related condition, and *SD*_*pi*_ is the pooled standard deviation of the two groups which is obtained through:

$${SD}_{pi} =\sqrt{{SD}_{1i}^{2}+ {SD}_{2i}^{2}-2{r}_{i}\times {SD}_{1i}{SD}_{2i}},$$

in which *n* is the number of participants, *SD*_1*i*_ is the standard deviation of RT in the control condition, and *SD*_2*i*_ is the standard deviation of RT in the phonologically related condition, *r*_*i*_ represents the correlation between RT in the two conditions. Given that correlations between the related and control conditions are rarely reported in priming studies, we assumed a moderate correlation between the two conditions (*r* = 0.5). To ensure the robustness of our results, we conducted a sensitivity analysis by varying *r* across different values (i.e., *r* = 0.1, *r* = 0.9; for the rationale, see Borenstein et al., 2009).

After some algebraic approximation procedures, *d*_*z*_ can also be calculated based on *t* statistic:

$${d}_{z} =t\sqrt{\frac{1}{\text{n}}} ,$$

or *F* statistic:

$${d}_{z}=\sqrt{\frac{F}{n}} ,$$

in which *n* is the number of participants, and the *t*-value and *F*-value are from the comparison between conditions by participants (see also Lucas, 2000; Van den Bussche et al., 2009; Wen & van Heuven, 2017). Therefore, for studies that did not report means and *SD*s of two conditions for effect size calculation while *t*- or *F*- statistics are available (i.e., Ando et al., 2014, 2015; Brysbaert et al., 1999; Chen et al., 2022, Experiment 1; Choi et al., 2010; Dimitropoulou et al., 2011; Kim et al., 2020; Nakayama et al., 2012), *d*_*z*_ is calculated in this way. Note that only* t* with a degree of freedom (*df*) of 1 or *F* with a denominator’s *df* of 1 was used to obtain Cohen’s *d*. For non-significant results where *t* or *F* statistics were not reported, we used the *p*-value to estimate the *t* or *F* values. If *p* was not provided in non-significant results (i.e., Experiment 3 of Gollan et al., 1997), we assumed *p* = 0.5 (Follmann et al., 1992; Frühauf et al., 2013).

The variance of *d*_*z*_ was calculated via:

$${v}_{i} =\frac{1}{n}+\frac{{{d}_{z}}^{2}}{2n}.$$

One study compared the cognate priming effect and noncognate priming effect to obtain the phonological priming effect. The *F*/*t* value did not have a *df* of 1. Therefore, we used the descriptive data of every participant to get the correlation between conditions. We used the following formula to get the priming effect:

$${d}_{rm} =\frac{{(M}_{RT1i}-{M}_{RT2i})-{(M}_{RT1i}-{M}_{RT3i}) }{SD},$$

where *d*_*rm*_ is another type of standardized difference score for a within-subject design (Lakens, 2013), obtained from the raw difference divided by the within-subject *SD*. ${M}_{RT1i}$ is the mean RT in the control condition, ${M}_{RT2i}$ is the mean RT in the noncognate condition, and ${M}_{RT3i}$ is the mean RT in the phonologically cognate condition. *SD* is the pooled standard deviation, which is calculated via the following formula:

$$SD=\sqrt{\frac{{s}_{gain}}{2(1-r)}},$$

where ${s}_{gain}$ is the standard deviation of gain scores, and *r* is the correlation between the cognate and noncognate priming effects. The variance of *d*_*rm*_ was obtained through:

$${v}_{i} =\frac{2\left(1-{r}_{1}\right)}{{n}_{1}}+\frac{2\left(1-{r}_{2}\right)}{{n}_{2}}+\frac{{{d}_{rm}}^{2}}{2\left({n}_{1}+{n}_{1}\right)}.$$

##### Subgroup analysis

We performed three subgroup analyses to examine the differences in priming effects between the two priming directions (from L1 to L2 and from L2 to L1), among the three tasks (lexical decision, word naming, and perceptual identification), and between the two types of script distance (within- and cross-script). Additionally, since the perceptual identification task only reported accuracy as the outcome, we conducted a subgroup analysis comparing the priming effects based on RT as the outcome to those calculated by accuracy.

#### Meta-regression analysis

The synthesized effects generally exhibit variability other than sampling errors, namely cross-effect heterogeneity. This heterogeneity affects the generalizability of our conclusions, and detecting its source can offer more insights into the mechanisms underlying the synthesized outcome (Deeks et al., 2019). We constructed a random-effects model to examine the role of various moderators. Since 16 studies reported multiple effects, there is a likelihood of dependency among them, a robust variance estimation (RVE; Hedges et al., 2010; for a review, see Fernández-Castilla et al., 2020) was employed to obtain unbiased nested-effects variance estimates for meta-analyses with a small number of included studies (less than 50; Moeyaert et al., 2017). We first examined the main effects of the seven moderators. Among them, three categorical variables, including priming direction, script distance, and task type, were included in the model as dummy-coded fixed effects, with lexical decision task, cross-script, and priming from L2 to L1 as reference groups. Note that the perceptual identification task was not included in the meta-regression analysis as a type of task due to a scarcity of effect sizes available (*k*_*2*_ = 6). The number of participants, the number of items, SOA, and ISI^2^ were continuous moderators in the random-effects model. We visually observed interaction patterns for all three categorical variables, i.e., the priming direction, task type, and script distance, when plotting their conditional means. However, no interaction pattern was found among the four continuous variables. Subsequently, we ran another model including the interaction terms between the three categorical moderators. Given the small number of within-script studies, we carried out the regression analysis with priming direction, task type, SOA, ISI, the number of participants, and the number of items as predictors in only cross-script studies.

The pooled cross-language phonological priming effect was to analyze the overall sample size regardless of conditions. The main effects model examined the role of each moderator without considering the interaction across moderators. The interaction effects model examined the interaction between task type and priming direction in addition to other factors since the interaction plot showed a trend of such interaction. The sensitivity analysis was to examine whether there were outlying effects or influential effects.

##### Sensitivity analysis

To test the robustness of the conclusion of this meta-analysis, we ran a group of sensitivity analyses. First, we left the outlying and influential effect sizes out one at a time (i.e., leave-one-out method, for an example, see Spence et al., 2021) to examine whether excluding each potentially outlying or influential effect leads to a different pattern of regression results. We identified effect size outliers and influential cases by calculating the external standardized residuals, hat values (*h*), Cook’s distance (*Cook’s D*), and DFBETAS using the *metafor* package (Viechtbauer, 2010), following the guidance in Viechtbauer and Cheung (2010). The external studentized residuals (also known as studentized deleted residuals) indicate the deviation of the observed effect size from the predicted value for the *i*th study based on the model without it. A large external studentized residual (e.g., exceeding an absolute value of 1.96) would indicate an outlying case. Regarding influential points, hat values indicate the influence of a case on the actual effect size, Cook’s distance measures the effect of deleting the *i*th effect on the fitted values of all *k*_*2*_ studies, and DFBETAS reveals the change of coefficients of each predictor once removing an effect. Then, we used the leave-one-out analysis to investigate the influence of potential outliers and influential effects on the regression outcome. We compared the between-study variance (*τ*^*2*^*)*, the proportion of *τ*^*2*^ changed in the deleted model (*R*^*2*^*)*, the variability among regression residuals (*Q*_*E*_*)*, and the reduced *Q*_*E*_ after deleting one effect in the regression (*ΔQ*_*E*_*)* of each deleted model (Hedges & Olkin, 2014; Viechtbauer & Cheung, 2010) to determine whether the effect is influential.

Since the correlation between related and control conditions is not known precisely when imputing the pooled standard deviation of the two groups, we tested with a range of plausible correlations and used a sensitivity analysis to see how different correlations impact estimating effect size *d*_*z*_ (Borenstein et al., 2009). Effect sizes were re-calculated and regression models were rerun after assigning different values (i.e., 0.1, 0.9) to correlation *r*.

#### Publication bias

**Fig. 2:**
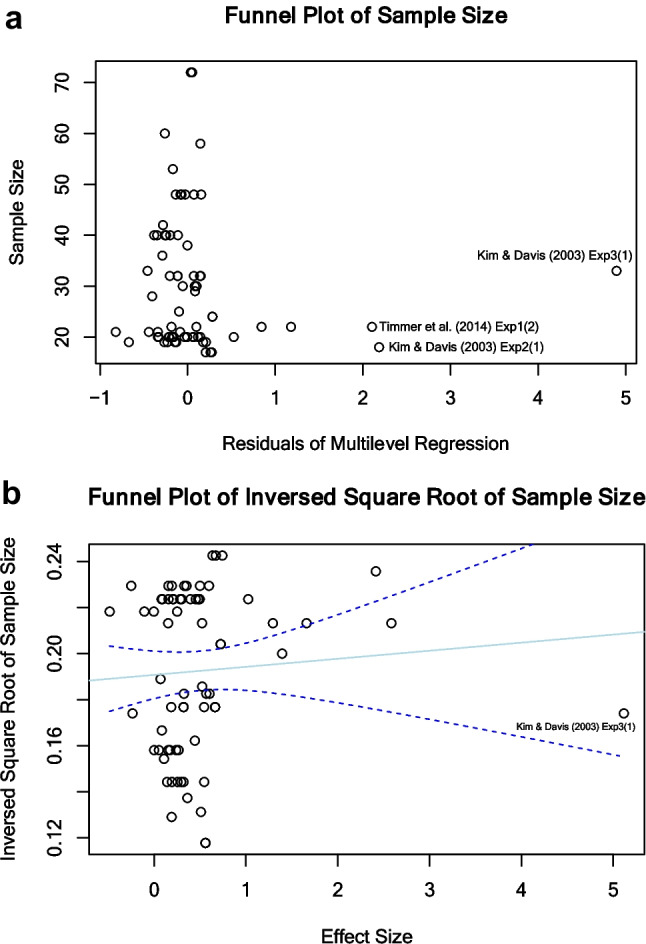
Enhanced contour funnel plot against sample size (**a**), and Regression plot of effect size against square root of inverse sample size (**b**)

To mitigate issues arising from potentially disproportionately reported findings (e.g., more significant facilitative phonological priming effects were reported in publications), we evaluated the possibility of publication bias in the included studies. We first utilized a funnel plot to visually inspect for small-study effects, which suggests that studies with a smaller sample size tend to exhibit larger effect sizes (Sterne & Egger, 2005). In the funnel plot (Fig. 2a), the residuals of a full multilevel meta-regression model were plotted against the sample sizes (Sterne and Egger, 2005; for an example, see Van den Bussche et al., 2009). Then, we executed a statistical test to further identify any evidence of publication bias. We used the multilevel meta-regression method (Nakagawa et al., 2022) in which the square root of the inverse sample size ($\sqrt{\frac{1}{n}}$) was included as a predictor in the full multilevel meta-regression model and the significance of the square root of inverse sample size was tested. Compared to the conventional Egger’s regression test, the multilevel meta-regression approach accounts for additional sources of variation in effect sizes (e.g., the moderators and random-effects predictors) and is less biased compared to a regression test using *SE*, precision, or variance as a moderator when using SMD as the effect size (for justification, see Nakagawa et al., 2022, Sect. 4.1; Doleman et al., 2020). The plot of effect sizes against the square root of inverse sample sizes is shown in Fig. 2b.

## Results

**Fig. 3:**
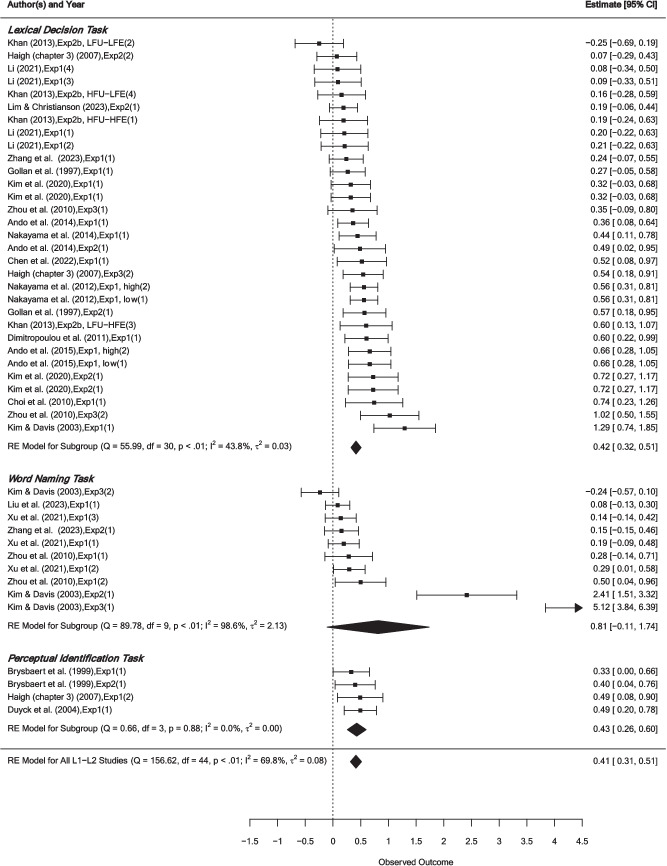
Forest plot for the effects of L1 to L2 priming

**Fig. 4:**
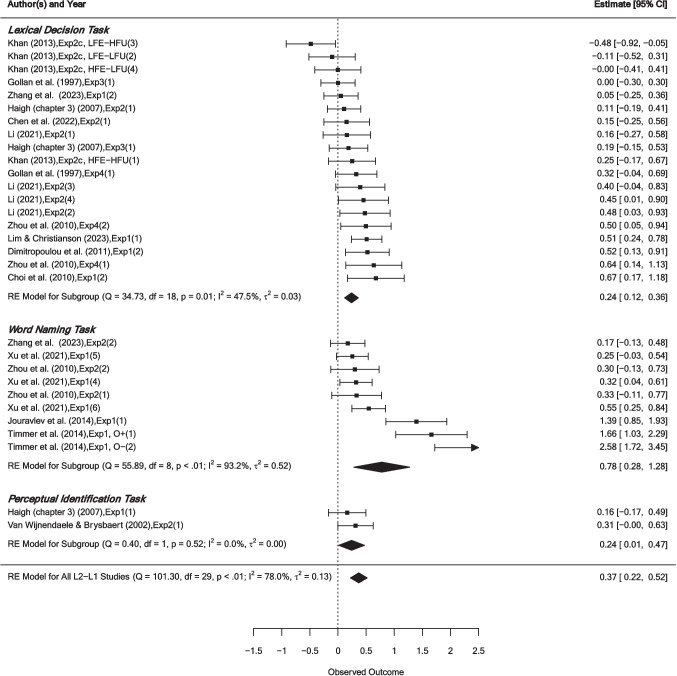
Forest plot for the effects of L2 to L1 priming

The SMD of included studies were summarized in two forest plots (Fig. 3 for priming effects from L1 to L2, and Fig. 4 for priming effects from L2 to L1).

### Descriptive statistics

Among the 75 effects derived from 23 studies, 45 effects tested the phonological priming effect from L1 to L2, and 30 tested it from L2 to L1. Fifty effects used a lexical decision task, 19 effects used a word-naming task, and six effects used a perceptual identification task. The languages of primes and targets used different scripts in 62 effects while 13 effects used the same script. Across all effects, the average number of participants was 30.75 (*SD* = 13.07), and the average number of items was 24.25 (*SD* = 33.28). The SOA was on average 72.82 (*SD* = 43.17), with an ISI of 14.66 (*SD* = 22.55). The characteristics of 23 cross-language phonological priming studies are described in Table 2 for L1-L2 effects and in Table 3 for L2-L1 effects.

**Table 2:** Description of 34 experiments extracted from 23 cross-script studies using masked phonological priming paradigm (Presented in Chronological Order)

Study	Experiment	Task	L1	L2	Dir	NoS	NoI	SOA	ISI	Target	RT_c	PE
Gollan et al. (1997)	Experiment 1	LDT	Hebrew	English	1	40	16	50	0	500	− 36	17^***^
	Experiment 2	LDT	English	Hebrew	1	30	16	50	0	500	− 52	90^**^
Brysbaert et al. (1999)	Experiment 1	PIT	Dutch	French	1	36	10	42	0	42	0.77	− 0.07^§^
	Experiment 2	PIT	Dutch	French	1	30	12	42	0	28	0.66	− 0.07^§^
Kim and Davis (2003)	Experiment 1	LDT	Korean	English	1	22	12	50	0	500	652	18
	Experiment 2	WNT	Korean	English	1	18	12	50	0	500	617	20^*^
	Experiment 3	WNT	Korean	English	1	33	10	50	0	500	645	24^*^
	Experiment 3, onset	WNT	Korean	English	1	33	10	50	0	500	645	− 1
Duyck et al. (2004)	Experiment 1	PIT	Dutch	French	1	50	10	42	0	42	0.82	− 0.07^§^ ^***^
Haigh (2008) (chapter 3)	Experiment 1	PIT	French	English	1	24	36	45	0	28	0.22	0.06^§^
	Experiment 2	LDT	French	English	1	28	36	45	0	/	812	5
	Experiment 3	LDT	French	English	1	32	36	250	0	/	733	30^*^
Zhou et al. (2010)	Experiment 1, high	WNT	Chinese	English	1	20	39	85	35	2000	631	10^**^
	Experiment 1, low	WNT	Chinese	English	1	19	39	85	35	2000	674	14^**^
	Experiment 3, high	LDT	Chinese	English	1	19	36	85	35	2000	666	14^***^
	Experiment 3, low	LDT	Chinese	English	1	20	36	85	35	2000	660	29^***^
Choi et al. (2010)	Experiment 1	LDT	Korean	English	1	17	34	90	40	2000	/	/^*^
Dimitropoulou et al. (2011)	Experiment 1	LDT	Greek	Spanish	1	30	30	50	0	2500	671	19^**^
Nakayama et al. (2012)	Experiment 1, low	LDT	Japanese (Katakana)	English	1	72	26	50	0	/	738	28^***^
	Experiment 1, high	LDT	Japanese (Katakana)	English	1	72	30	50	0	/	674	31^***^
Khan (2013)	Experiment 2b, HFU-HFE	LDT	Urdu	English	1	19	5	50	0	/	11	42.6
	Experiment 2b, LFU-LFE	LDT	Urdu	English	1	19	5	50	0	/	− 47.2	− 41.7
	Experiment 2b, LFU-HFE	LDT	Urdu	English	1	19	5	50	0	/	66.5	55.9^*^
	Experiment 2b, HFU-LFE	LDT	Urdu	English	1	19	5	50	0	/	25.2	29.8
Ando et al. (2014)	Experiment 1	LDT	Japanese (Kanji)	English	1	53	32	50	0	1500	728	17^**^
	Experiment 2	LDT	Japanese (Kanji)	English	1	20	30	50	0	2500	615	18^*^
Nakayama et al. (2014)	Experiment 1	LDT	Japanese (Katakana)	English	1	38	23	50	0	2000	72	− 47^***^
Ando et al. (2015)	Experiment 1, low	LDT	Japanese (Katakana)	English	1	32	28	50	0	1500	872	20^***^
	Experiment 1, high	LDT	Japanese (Katakana)	English	1	32	30	50	0	1500	798	33^***^
Kim et al. (2020)	Experiment 1, full overlap	LDT	Korean	English	1	32	10	50	0	3000	745	14^†^
	Experiment 1, partial overlap	LDT	Korean	English	1	32	10	50	0	3000	745	14^†^
	Experiment 2, full overlap	LDT	Korean	English	1	24	10	40	0	3000	730	52^***^
	Experiment 2, partial overlap	LDT	Korean	English	1	24	10	40	0	3000	730	52^***^
Xu et al. (2021)	Experiment 1, SOA 43	WNT	Chinese	English	1	50	10	43	0	3000	648	9
	Experiment 1, SOA 75	WNT	Chinese	English	1	50	10	75	0	3000	649	14^*^
	Experiment 1, SOA 150	WNT	Chinese	English	1	50	10	100	0	3000	659	7^†^
Li (2021)	Experiment 1, HF-HP	LDT	Chinese	English	1	20	16.75	85	35	2000	575	11^†^
	Experiment 1, LF-HP	LDT	Chinese	English	1	20	16.75	85	35	2000	726	20^†^
	Experiment 1, HF-LP	LDT	Chinese	English	1	20	16.75	85	35	2000	634	7
	Experiment 1, LF-LP	LDT	Chinese	English	1	20	16.75	85	35	2000	803	9
Chen et al. (2022)	Experiment 1	LDT	Chinese	English	1	25	40	100	100	1500	− 60	23.5^*^
Lim and Christianson (2023)	Experiment 2	LDT	Korean	English	1	60	12	50	0	/	869	14.1
Liu et al. (2023)	Experiment 1	WNT	English	Japanese (Kanji)	1	36	50	58.3	8.3	3000	− 48	5.87
Zhang et al. (2024)	Experiment 1	LDT	Chinese	English	1	40	38	90	50	2000	739	16^***^
	Experiment 2	WNT	Chinese	English	1	40	38	90	50	2000	810	5^*^

*Dir*, priming direction; *NoP*, number of participants; *NoI*, number of items per cell; *Target*, target duration; *RT-c*, reaction times in the control conditions; *PE*, priming effects

^§^ Studies used PIT reported response accuracy rather than response latency for the priming effect

^†^*p* <.10, ^*^p <.05, ^**^p <.01, ^***^p <.001

LDT = Lexical decision task, WNT = Word naming task, PIT = Perceptual identification task. Priming effect (PE) was the difference between the response latency/accuracy in the related condition and that in the unrelated condition. “/” in the target duration means the target stayed on the screen until a response was made

**Table 3:** Description of 20 experiments extracted from 15 within-script studies using masked L2-L1 phonological priming paradigm (Presented in Chronological Order)

Study	Experiment	Task	L1	L2	Dir	NoS	NoI	SOA	ISI	Target	RT_c	PE
Gollan et al. (1997)	Experiment 3	LDT	English	Hebrew	2	40	16	50	0	500	− 9	0
	Experiment 4	LDT	Hebrew	English	2	30	16	50	0	500	4	8^†^
van Wijnendaele and Brysbaert (2002)	Experiment 2	PIT	French	Dutch	2	39	12	42	0	28	0.58	− 0.06^§*^
Haigh (2008) (chapter 3)	Experiment 1	PIT	English	French	2	34	36	45	0	28	0.26	0.03^§^
	Experiment 2	LDT	English	French	2	42	36	45	0	/	673	5
	Experiment 3	LDT	English	French	2	32	36	250	0	/	671	8
Zhou et al. (2010)	Experiment 2, high	WNT	Chinese	English	2	19	39	85	35	2000	551	7^*^
	Experiment 2, low	WNT	Chinese	English	2	20	39	85	35	2000	594	9^*^
	Experiment 4, high	LDT	Chinese	English	2	17	40	85	35	2000	599	25^**^
	Experiment 4, low	LDT	Chinese	English	2	20	40	85	35	2000	544	10^**^
Choi et al. (2010)	Experiment 1	LDT	Korean	English	2	17	34	90	40	2000	/	/^*^
Dimitropoulou et al. (2011)	Experiment 1	LDT	Greek	Spanish	2	29	30	50	0	2500	645	15^**^
Khan (2013)	Experiment 2c, HFE-HFU	LDT	Urdu	English	2	21	5	50	0	/	51.5	59.5
	Experiment 2c, LFE-LFU	LDT	Urdu	English	2	21	5	50	0	/	15.7	− 35.2
	Experiment 2c, LFE-HFU	LDT	Urdu	English	2	21	5	50	0	/	− 31	− 143^*^
	Experiment 2c, HFE-LFU	LDT	Urdu	English	2	21	5	50	0	/	102	− 1.09
Jouravlev et al. (2014)	Experiment 1	WNT	Russian	English	2	25	288	48	17	2000	643	23^***^
Timmer et al. (2014)	Experiment 1, O +	WNT	Dutch	English	2	22	38	48	17	2000	577	9
	Experiment 1, O-	WNT	Dutch	English	2	22	38	48	17	2000	581	14
Li (2021)	Experiment 2, HF-HP	LDT	Chinese	English	2	20	16.75	85	35	2000	632	13
	Experiment 2, LF-HP	LDT	Chinese	English	2	20	16.75	85	35	2000	764	59^**^
	Experiment 2, HF-LP	LDT	Chinese	English	2	20	16.75	85	35	2000	784	50^***^
	Experiment 2, LF-LP	LDT	Chinese	English	2	20	16.75	85	35	2000	932	68^**^
Xu et al. (2021)	Experiment 1, SOA 43	WNT	Chinese	English	2	50	10	43	0	3000	598	14^†^
	Experiment 1, SOA 75	WNT	Chinese	English	2	50	10	75	0	3000	607	11
	Experiment 1, SOA 150	WNT	Chinese	English	2	50	10	100	0	3000	641	24^*^
Chen et al. (2022)	Experiment 2	LDT	Chinese	English	2	25	40	100	100	1500	− 43.2	10.8
Lim and Christianson (2023)	Experiment 1	LDT	Korean	English	2	58	12	50	0	/	867	71.5^*^
Zhang et al. (2023)	Experiment 1	LDT	Chinese	English	2	40	38	90	50	2000	574	2
	Experiment 2	WNT	Chinese	English	2	40	38	90	50	2000	694	4

*Dir*, priming direction; *NoP*, number of participants; *NoI*, number of items per cell; *RT-c*, reaction times in the control conditions; *PE*, priming effects

^§^ Studies used PIT reported response accuracy rather than response latency for the priming effect

^†^*p* <.10, ^*^p <.05, ^**^p <.01, ^***^p <.001

LDT = Lexical decision task, WNT = Word naming task, PIT = Perceptual identification task. Priming effect (PE) was the difference between the response latency/accuracy in the related condition and that in the unrelated condition. “/” in the target duration means the target stayed on the screen until a response was made

### Pooled cross-language phonological priming effect

The mean effect size for the random-effects model was *g* = 0.45, *SE* = 0.07, *p* < 0.0001, 95% CI = [0.32, 0.58], indicating a significant and facilitative phonological priming effect. In other words, seeing a phonologically related prime accelerates the processing of the target. The amount of between-effects heterogeneity was estimated to be 0.09 (*SE* = 0.29). The analysis of the null model showed significant cross-effect heterogeneity, with *Q*(74) = 259.40, *p* < 0.0001, suggesting that there are potential factors responsible for the variance among effects beyond sampling errors.

#### Subgroup analysis

##### Priming direction

The average priming effect size from L1 to L2 was significant and facilitative *g* = 0.41, *SE* = 0.05, *k*_*2*_ = 45, *p* < 0.0001, 95% CI = [0.31, 0.51]. The average priming effect size from L2 to L1 was significant and facilitative, *g* = 0.37, *SE* = 0.08, *k*_*2*_ = 30, *p* < 0.0001, 95% CI = [0.22, 0.52]. Priming studies in both directions showed a significant amount of heterogeneity: from L1 to L2, *τ*^*2*^ = 0.08, *I*^*2*^ = 69.78%, *Q*(44) = 156.62, *p* < 0.0001; from L2 to L1, *τ*^*2*^ = 0.13, *I*^*2*^ = 77.97%, *Q*(29) = 101.30, *p* < 0.0001.

##### Script distance

Both within- and cross-script had significant, facilitative phonological priming effects. The within-script phonological priming was *g* = 0.50, *SE* = 0.16, *k*_*2*_ = 13, *p* = 0.002, 95% CI = [0.19, 0.80]; the cross-script phonological priming was *g* = 0.38, *SE* = 0.04, *k*_*2*_ = 62, *p* < 0.0001, 95% CI = [0.30, 0.47]). Both within- and cross-script priming studies yielded a significant amount of heterogeneity: within-script, *τ*^*2*^ = 0.28, *I*^*2*^ = 90.10%, *Q*(12) = 57.07, *p* < 0.0001; cross-script, *τ*^*2*^ = 0.08, *I*^*2*^ = 68.88%, *Q*(61) = 202.09, *p* < 0.0001.

##### Task type

The average priming effect size of the lexical decision task was significant and facilitative, *g* = 0.35, *SE* = 0.04, *k*_*2*_ = 50, *p* < 0.0001, 95% CI = [0.27, 0.43]. A significant amount of heterogeneity existed among effects using a lexical decision task, *τ*^*2*^ = 0.04, *I*^*2*^ = 50.83%, *Q*(49) = 100.63, *p* < 0.0001. The average priming effect size of the word-naming task was significant and facilitative, *g* = 0.78,^3^*SE* = 0.25, *k*_*2*_ = 19, *p* = 0.002, 95% CI = [0.29, 1.28]. A significant amount of heterogeneity was observed among effects using a word-naming task, *τ*^*2*^ = 1.16, *I*^*2*^ = 97.26%, *Q*(18) = 155.76, *p* < 0.0001. Furthermore, the average priming effect size of the perceptual identification task was significant and facilitative, *g* = 0.36, *SE* = 0. 07, *k*_*2*_ = 6, *p* < 0.0001, 95% CI = [0.23, 0.50], but the heterogeneity among effects was not significant, *τ*^*2*^ = 0, *I*^*2*^ = 0, *Q*(5) = 2.72, *p* = 0.743. Due to a very small number of effects available using the perceptual identification task, we were not able to include this task in the regression analysis.

### Meta-regression

We conducted the meta-regression test to examine how moderators explain the heterogeneity across effects. We used the *rma.mv*() and *robust.rma.mv*() functions in the *metafor* package (Viechtbauer, 2010) in R (R Core Team, 2024) to obtain a robust estimation of coefficients. See Table 4 for the meta-regression analysis results.

**Table 4:** Meta-regression analysis results with (**a**) and without (**b)** potential outliers and influential effects

					*b*			*SE*	95% CI			*t*			*df*			*p*
(a) Regression Analysis Results																		
Intercept					0.40			0.08	(0.23, 0.57)			5.04			14			<.001^***^
Priming direction					− 0.09			0.09	(− 0.29, 0.12)			− 0.90			14			.381
Task					− 0.22			0.11	(− 0.46, 0.02)			− 1.98			14			.067^†^
ISI					− 0.001			0.002	(− 0.005, 0.002)			− 0.90			14			.381
N of Items					0.004			0.001	(0.003, 0.005)			6.44			14			<.0001^***^
Priming direction × Task					0.25			0.12	(− 0.01, 0.52)			2.03			14			.061^†^
(b) Regression Analysis Results (Excluding Effects #11, 20, 21)																		
Intercept		0.35		0.06			(0.22, 0.48)				5.79			13			<.0001^***^	
Prime direction		− 0.11		0.10			(− 0.32, 0.09)				− 1.18			13			.260	
Task		− 0.29		0.09			(− 0.49, − 0.1)				− 3.23			13			.007^**^	
ISI		− 0.001		0.001			(− 0.004, 0.002)				− 1.06			13			.308	
N of Items		0.004		0.001			(0.003, 0.006)				8.35			13			<.0001^***^	
Priming direction × Task		0.25		0.11			(0.01, 0.49)				2.24			13			.043^*^	
(c) Regression Analysis Results for Cross-Script Studies (Excluding Effects #11, 20, 21)																		
Intercept	0.36		0.06			(0.22, 0.50)				5.58			11			<.001^***^		
Prime direction	− 0.10		0.11			(− 0.34, 0.15)				− 0.87			11			.404		
Task	− 0.25		0.09			(− 0.45, − 0.05)				− 2.82			11			.017^*^		
ISI	− 0.002		0.002			(− 0.005, 0.001)				− 1.29			11			.223		
N of Items	0.005		0.001			(0.003, 0.006)				6.90			11			<.0001^***^		
Priming direction × Task	0.21		0.13			(− 0.08, 0.49)				1.61			11			.136		

^†^*p* <.10, ^*^p <.05, ^**^p <.01, ^***^p <.001

Explanation of coefficients: priming direction = L1-to-L2 – L2-to-L1, Task = word-naming task—lexical decision task

#### Main effects model

The best main effects model included moderators priming direction, script distance, task type, ISI, the number of participants, and the number of items (*AICc* = 120.68). The number of items had a significant, positive impact on effect sizes, *b* = 0.004, *SE* = 0.001, *t*(13) = 4.98, *p* = 0.0003, indicating that the more items tested in the experiment, the larger the effect size was. All other moderators were not significant (*p*s > 0.119) in the main effects model.

#### Interaction effects model

From the interaction plot, the three categorical variables, i.e., priming direction, task type, and script distance, showed a trend of interaction. However, after the perceptual identification task was removed from regression analysis, there were only seven effects left in the within-script category, resulting in an insufficient number of effects per cell for a robust by-group comparison analysis on this level. Therefore, script distance could not be included in the interaction terms, leaving only the interaction between priming direction and task type in the interaction effects model. The ultimate interaction model contains the main effects of priming direction, task type, number of items in each condition, ISI, and an interaction term between priming direction and task type (*AICc* = 123.64, *R*^*2*^ = 33.44%). The inclusion of any other variables (script distance, the number of participants, and SOA) resulted in a decrease in the variance explained by the model. In the interaction model, the number of items remained a significant, positive moderator, *b* = 0.004, *SE* = 0.001, *t*(14) = 6.44, *p* < 0.001. In addition, task type was marginally significant, *b* = − 0.22, *SE* = 0.11, *t*(14) = − 1.98, *p* = 0.067; the interaction between priming direction and task type was marginally significant, *b* = 0.25, *SE* = 0.12, *t*(14) = 2.03, *p* = 0.061. The other moderators were non-significant (*p*s > 0.381). Results are summarized in Table 4a.

#### Absolute differences in milliseconds

Considering the potential inconsistencies introduced by various types of SMD (Thom, 2009; Pek & Flora, 2018), we conducted additional analyses using absolute differences in milliseconds (*M* = 16.26, 95% CI = [9.11, 23.41]). This approach enabled us to examine whether the conclusions drawn from standardized effect sizes align with those based on raw difference scores. Absolute differences were used as the outcome variable, with priming direction, script distance, task type, the interaction between priming direction and task type, SOA, and the number of items, entered as predictors in a linear mixed-effects model. Results indicated that none of the predictors were significant, *p*s > 0.399.

#### Sensitivity analysis

**Fig. 5:**
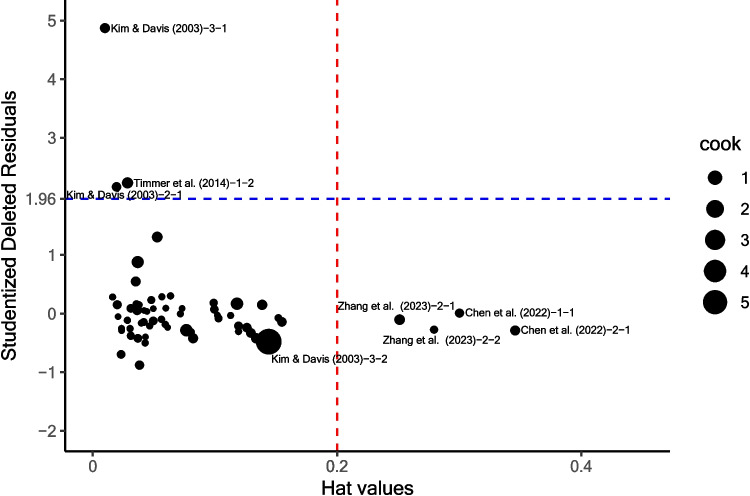
Diagnostic plot of multilevel meta-regression. Note. Jouravlev et al. (2014) was not shown in this plot due to the extreme hat value and Cook’s distance (h = 0.94, Cook’s D = 21.8, residual = − 1.86)

**Fig. 6:**
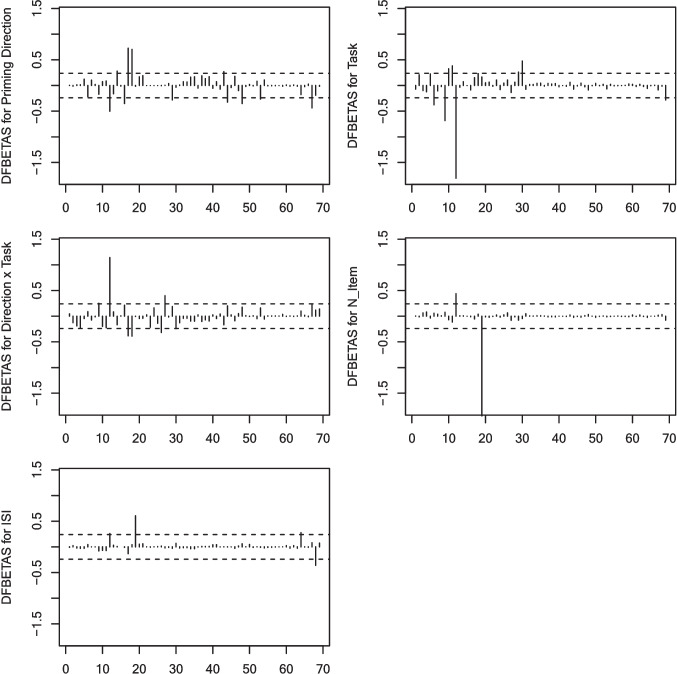
DFBETAS for each predictor in meta-regression

A model diagnosis was run based on the interaction model. The diagnostic result is plotted in Fig. 5 (external studentized residuals, hat values, and Cook’s distance) and Fig. 6 (DFBETAS). Using 1.96 as the absolute cutoff value, we found six effects as outliers (three effects in Kim & Davis, 2003; one effect in Khan, 2013; two effects in Timmer et al., 2014). Using 0.2 as the cutoff value for hat values and ${\chi }_{5, 0.5}^{2}$ = 4.35 as the cutoff value for Cook’s distances, nine effects were found to be potentially influential (i.e., three effects in Kim & Davis, 2003; two effects in Chen et al., 2022; one effect in Jouravlev et al., 2014; one effect in Timmer et al., 2014; two effects in Zhang et al., 2024). DFBETAS suggested that 22 effects altered the coefficient estimates of at least one predictor to a large degree (> 0.241^4^; Fig. 6). Among all potential outliers and influential effects that were further checked in the leave-one-out analysis, only effects #11 (Kim & Davis, 2003, Experiment 3), #20 (Timmer et al., 2014, Experiment1 (1)), and #21 (Timmer et al., 2014, Experiment 1 (2)) significantly impacted the regression results in several ways. Before these effects was excluded, both the effects of task type and the number of items were marginally significant; however, upon its removal, they became statistically significant. After the exclusion, the effect sizes for L1-to-L2 cross-script studies were *g* = 0.42 (*SE* = 0.05) for the LDT and *g* = 0.38 (*SE* = 0.21) for the naming task. Moreover, effects #20 and 21 have a large positive reduced *R*^*2*^ (57.37% and 60.68%, respectively, compared to other effects that have smaller reduced *R*^*2*^ < 13.48^5^); effects #11 and 21 have a large *ΔQ*_*E*_ (23.23 and 58.01, respectively, compared to other effects that have smaller *ΔQ*_*E*_ < 13.73), suggesting that the inclusion of these three effects substantially increased between-study variability and regression residuals. The $\tau$ ^*2*^, reduced *R*^*2*^, *Q*_*E*_, and *ΔQ*_*E*_ of the deleted models are summarized in Table 5.

**Table 5:** Results for leave-one-study out analysis

Effect No	Experiment	*τ*^*2*^	Changed *R*^*2*^* (%)*	*QE*	*ΔQE*
6	Zhou et al. (2010) Exp3 (2)	0.072	4.03	212.66	5.03
9	Kim and Davis (2003) Exp1 (1)	0.065	13.48	207.33	10.36
10	Kim and Davis (2003) Exp2 (1)	0.066	12.1	193.72	23.97
11	Kim and Davis (2003) Exp3 (1)	0.065	13.4	159.68	**58.01**
12	Kim and Davis (2003) Exp3 (2)	0.220	− 192.58	211.6	6.09
14	Haigh (2007) Exp2 (2)	0.072	3.9	214.03	3.66
16	Haigh (2007) Exp3 (2)	0.081	− 7.54	217.18	0.51
17	Lim and Christianson (2023) Exp1 (1)	0.080	− 6.37	213.02	4.67
18	Lim and Christianson (2023) Exp2 (1)	0.079	− 4.5	215.09	2.6
19	Jouravlev et al. (2014) Exp1 (1)	0.086	− 13.93	216.53	1.16
20	Timmer et al. (2014) Exp1 (1)	0.032	**57.37**	203.97	13.72
21	Timmer et al. (2014) Exp1 (2)	0.030	**60.68**	194.46	**23.23**
26	Xu et al. (2021) Exp1 (5)	0.073	3.48	216.42	1.27
27	Xu et al. (2021) Exp1 (6)	0.079	− 5.22	216.5	1.19
29	Zhang et al. (2024) Exp1 (2)	0.079	− 4.25	215.75	1.94
30	Zhang et al. (2024) Exp2 (1)	0.081	− 7.52	217.59	0.1
31	Zhang et al. (2024) Exp2 (2)	0.073	3.51	214.26	3.43
43	Khan (2013) Exp1 (2)	0.071	6.28	209.98	7.71
44	Khan (2013) Exp1 (3)	0.082	− 8.58	216.69	1
48	Khan (2013) Exp2 (3)	0.071	6.06	207.72	9.97
53	Gollan et al. (1997) Exp2 (1)	0.079	− 4.97	217	0.69
64	Chen et al. (2022) Exp1 (1)	0.077	− 2.53	216.87	0.82
67	Gollan et al. (1997) Exp3 (1)	0.074	1.23	214.88	2.81
68	Chen et al. (2022) Exp2 (1)	0.078	− 3.28	217.67	0.02
69	Liu et al. (2023) Exp1 (1)	0.074	2.35	211.48	6.21

*τ*_*0*_^*2*^ = 0.075, *QE*_*0*_ = 217.69 for the original full model. *ΔQE*_*i*_ = *QE*_*0*_* – QE*_*i*_,

$$Changed {R}^{2}=\frac{\left({\tau }_{0}^{2}-{\tau }_{-i}^{2}\right)}{{\tau }_{0}^{2}}$$

Effects #11, 20, and 21 are concerning due to a high positive changed *R*^*2*^ and/or a high *ΔQE* after deleting it.

Here are the results after excluding effects #11, 20, and 21 (*k*_*2*_ = 66; Table 4b). The interaction between task type and priming direction was significant, *b* = 0.25, *SE* = 0.11, *t*(13) = 2.24, *p* = 0.043. For priming direction from L1 to L2, the lexical decision task yielded a larger effect than the word-naming task, *b* = − 0.29, *SE* = 0.09, *t*(13) = − 3.23, *p* = 0.007; for priming direction from L2 to L1, there was no significant difference between the two tasks (*p* = 0.691). The number of items in each condition had a significant, positive effect on the size of the phonological priming effect, *b* = 0.004, *SE* = 0.001, *t*(13) = 8.37, *p* < 0.0001. The more items, the larger the positive priming effect. No significant difference was observed across different priming direction and ISI conditions (*p*s > 0.259).

Given the notable imbalance between within-script studies (*k*_*2*_ = 5) and cross-script studies (*k*_*2*_ = 61), we ran another regression model with only cross-script studies to determine whether the results were predominantly driven by cross-script studies. In this model, the interaction between priming direction and task type was not significant (*p* = 0.136). The main effect of task type was significant, the priming effect in the lexical decision task was significantly larger than that in the word-naming task, *b* = − 0.25, *SE* = 0.09, *t*(11) = − 2.82, *p* = 0.017. In addition, the number of items in each condition had a significant positive effect on the size of the phonological priming effect, *b* = 0.005, *SE* = 0.001, *t*(11) = 6.90, *p* < 0.0001. No significant effect was found for priming direction or SOA (*ps* > 0.223).

A sensitivity analysis was conducted to test the robustness of the estimated effect size *d*_*z*_ (Borenstein et al., 2009). We calculated effect sizes and ran regression models while manipulating *r* at 0.1 and 0.9, the estimates of effect sizes remained similar. This suggests that the results generated under the default *r* value (0.50) for computing within-study effect sizes were robust and reliable. Therefore, we reported analyses based only on the value of *r* = 0.50.

### Publication bias

The multilevel regression test in which the square root of inverse sample size ($\sqrt{\frac{1}{n}}$) was included as a predictor in the full multilevel meta-regression model did not reveal any significant asymmetry (*p* = 0.764), indicating that this meta-analysis is unlikely to be subject to publication bias.

## Discussion

This meta-analysis systematically reviewed empirical evidence on cross-language phonological priming in the literature and investigated the influence of multiple potential moderators. Based on a large number of participants (*n* = 1,387), we observed significant phonological priming effects across two priming directions (from L1 to L2 or from L2 to L1), three tasks (lexical decision, word naming, or perceptual identification), and two types of script distance (within- or cross-script). We conducted meta-regression analyses to investigate the effects of seven potential moderators on the strength of these priming effects. After excluding an influential effect, the lexical decision task yielded a larger effect than the word-naming task in cross-script studies. We also observed a positive relationship between the effect size and the number of items. The more items included, the stronger the effect. Taken together, our review is among the first to use the meta-analytic approach to show a robust cross-language phonological priming under different conditions, supporting the hypothesis of shared phonological representation in the integrated bilingual lexicon and the view of nonselective phonological activation. Our results also underscore the significant roles of task demands and the number of items in cross-language phonological activation. The implications of these findings are further discussed in the following sections.

### Cross-language phonological activation

How does cross-language phonological priming happen? The process can be twofold: first, access to the prime in one language activates the phonological representation shared by two languages; second, the activated shared phonological representation enhances the recognition of the target word in the other language (Brysbaert, 2003; Desmet & Duyck, 2007; Jiang, 2023). The significant priming effect suggests that the phonological representations of two languages are stored in the same or adjacent space in a bilingual lexicon (Francis, 2005). This way, readers do not selectively activate the phonological information in one language, even when the target language is specified in the task (e.g., Carrasco‐Ortiz et al., 2012). Additionally, the phonological priming is indeed largely contingent upon the activated phonological representation in both L1 and L2, since the orthographic or semantic overlap has been reduced to a large degree in empirical studies. Our meta-analysis showcased an automatic phonological activation that occurs not only during L1 visual word recognition but also during L2 visual word recognition. Take priming from L1 to L2 as an example. On one hand, only if the phonological representation of the L1 prime is activated can it influence the processing of the L2 target word, increasing the activation level of its homophone (i.e., the target) from resting, and enhancing the recognition of the target word. On the other hand, if the processing of the target bypasses phonological activation, it would not benefit from the co-activated phonological information from the prime. Note that all studies included in the current meta-analysis used rigorous masking design, ensuring subliminal processing and minimizing the strategic processing due to participants’ awareness of phonological overlap. Therefore, the significant priming synthesized in our study provides rigorous evidence for automatic phonological activation in bilingual lexical processing.

The existence of automatic phonological activation in reading provides additional evidence in favor of the strong phonological view. Our result aligns with the finding from a meta-analysis on phonological preview benefit effect (Vasilev et al., 2019), where a modest but reliable effect was revealed: fixation durations are shorter when participants have a valid preview of the target word (e.g., “bear”) compared to when such preview is denied (e.g., the word “bear” is masked by a string of Xs).

The finding of cross-language phonological priming is consistent with the architecture of the bilingual lexicon proposed in the BIA + model and the Multilink model. In both models, lexical representations of two languages are stored in the same location at both lexical and sublexical levels, regardless of the language. The determination of language membership and the subsequent processing are managed solely by a language node. Particularly noteworthy is the use of shared sublexical and lexical phonological representations by the two languages known to a bilingual. In a phonological priming trial, the common sublexical units are activated during the processing of the prime. The target recognition is enhanced through feedback from the co-activated sublexical phonological units to the lexical phonological units, and to orthographic representations at both sublexical and lexical levels. In addition to the common phonological units, some adapted versions of models of BIA + (e.g., Ando et al., 2014; Miwa et al., 2014; Jiang, 2023) also mentioned phonological units unique to each language, which do not have a close approximation in the other language, such as the diphthong/ɔɪ/and the voiced dental fricative/ð/in English that do not exist in Chinese for a Chinese-English bilingual. Yet, how these language-specific phonological units would affect cross-language phonological priming is unclear based on these models. Given that previous research has heavily focused on the shared phonological units among prime-target pairs across two languages, future research is warranted to involve the language-specific phonological units in prime-target pairs so as to form direct comparisons to those pairs with shared units.

### The role of task type

Our results showed that the effect of task type was significant in cross-script phonological priming, with the lexical decision task exhibiting a larger effect than the word-naming task. This finding contradicts some earlier monolingual studies (e.g., Coltheart, 1985; De Groot et al., 2002; Shen & Forster, 1999), which suggested a larger phonological priming effect in the word-naming task than in the lexical decision task. These studies indicated that during a lexical decision task, the process of differentiating real words from non-words may draw upon phonological, orthographic, or semantic representations, and thus reduces the necessity for explicit phonological access (De Groot et al., 2002; Dijkstra & van Heuven, 2002; Kim & Davis, 2003). Conversely, the word-naming task integrates word recognition with oral production, wherein the latter step mandates the engagement of phonological information. Hence, greater importance is assigned to the phonological code in the word-naming task, presumably leading to a heightened incidence of phonological priming effects.

However, bilingual studies on cross-language phonological priming effects have produced mixed results regarding task-dependent effects. For instance, Kim and Davis (2003) found no significant phonological priming effect from L1 to L2 in the lexical decision task. They speculated that this lack of priming effect could stem from participants’ preference for orthographic and semantic processing over phonological activation, which is particularly likely with primes being masked. Thus, the phonological relation between primes and targets is not consciously perceived and utilized. However, this study was not only later critiqued for its small sample size but also contradicted by subsequent studies that incorporated both lexical decision and word-naming tasks. Those studies reported significant phonological priming in both tasks (Zhang et al., 2024; Zhou et al., 2010), with a larger priming effect size in the lexical decision task.

The larger phonological priming in lexical decision tasks observed in our analysis probably stems from the need for bilinguals to modulate cross-language activity to prioritize the target language while inhibiting responses in the non-target language (Kroll et al., 2008). During word naming, especially when there is masked phonological priming, the primary mechanism is linked to the articulatory processes rather than direct lexical access (Shen & Forster, 1999). The word-naming task requires a higher level of precision for phonological representations, compared with the lexical decision. As a result, the phonological priming in word naming is reduced by substantial inhibition of non-target language phonological representation, even though both tasks leverage phonological similarities to enhance the activation level of corresponding representations across languages. The variation in articulatory inhibition across languages thus potentially accounts for the discrepancies observed between monolingual (e.g., De Groot et al., 2002; Shen & Forster, 1999) and bilingual (e.g., Zhang et al., 2024; Zhou et al., 2010) research.

The greater inhibitory demand on articulation aligns with findings in code-switching research, where bilinguals switch languages promptly which entails a cognitive cost. A comparison of switch costs under different conditions shows that they are generally lower in comprehension tasks (e.g., lexical decision; Aparicio & Lavaur, 2014; Orfanidou & Sumner, 2005; Struck & Jiang, 2022; Thomas & Allport, 2000; Von Studnitz & Green, 1997), as opposed to production tasks requiring articulation (e.g., picture or numeric naming; Christoffels et al., 2007; Costa & Santesteban, 2004; Declerck et al., 2012, 2013; Hernandez & Kohnert, 1999; Meuter & Allport, 1999; Philipp et al., 2007; Prior & Gollan, 2011; Zheng et al., 2020). Language comprehension requires less rigorous phonological discrimination across languages, thus showing a reduced need for inhibition. Accordingly, the cross-language inhibition effect is smaller in lexical decision, a potential reason for the larger cross-language phonological priming effect shown in the current meta-analysis.

The effect of task type shown in our study supports the necessity of the task schema proposed in the BIA + model and the Multilink model. The task schema can modulate the weights of different lexical representations including varying weights of phonological representation in different tasks. Our finding supports this proposal that when a task is phonologically more demanding (e.g., word-naming task in this review), heavier weights on phonological representation are assigned, leading to a stronger need for inhibition of phonological representation across the languages. As a result, a smaller phonological priming effect occurs. Altogether, our finding regarding the task effect demonstrates the importance of considering task demand in cross-language phonological priming research, pointing to the multitude of cognitive processes employed in bilingual processing.

### The role of priming direction

Brysbaert (2003) pointed out that homophones in L1 and L2 could prime each other based on three empirical studies (i.e., Brysbaert et al., 1999; Brysbaert & Van Wijnendaele, 2003; van Wijnendaele & Brysbaert, 2002). Since then, the number of studies on cross-language phonological priming in two different priming directions has increased, but still with a preference for priming from L1 to L2. To the best of our knowledge, this meta-analysis is the first to directly compare a large sample of phonological priming effects in the two directions. Although the L1-to-L2 priming showed a trend to be greater than the L2-to-L1 priming, the asymmetric pattern was not significant. This finding suggests that the shared phonological representation probably has a single representation code for two languages, providing strong evidence for an integrated lexicon regardless of language dominance.

Our finding contrasts with previous research that identified an asymmetric translation priming effect, with priming from L1 to L2 appearing to be significantly stronger than that from L2 to L1 among unbalanced bilinguals (Schoonbaert et al., 2009; Wen & van Heuven, 2017; Zhao & Li, 2013). A widely accepted explanation is that the ways L1 and L2 representations are activated differ quantitatively, as suggested by several bilingual models. For instance, L2 words activate fewer semantic features than L1 words do (e.g., the Sense model by Finkbeiner et al., 2004, and the Distributed Representation Model by van Hell and de Groot, 1998), which leads to overall weaker activation of semantic features of these L2 words (Duyck et al., 2004). Alternatively, semantic representations in L2 may be activated at a slower speed than those in L1, causing a stronger cross-linguistic influence from L1 primes to L2 targets than in the reverse direction (e.g., the BIA + model by Dijkstra & van Heuven, 2002).

This explanation attributes the asymmetry in semantic priming to the different numbers and activation strengths of the associations between lexical representations and semantic features across languages. In contrast, cross-language phonological priming hinges on shared phonological representation (see Jiang, 2023). On one hand, words in either language have a definite number of phonological units, which are usually comparable across interlingual homophones used in the reviewed studies (e.g., the French word *oui* and the English word *we* both have two phonemes). Therefore, the activation of more or fewer features no longer holds in phonological priming. On the other hand, the absence of phonological priming asymmetry in our analysis may suggest that there is a comparable strength in the connections between orthography and phonology in both languages. This means that the possibility of activation from orthography to phonology in either dominant or nondominant language is similar. Our finding offers a novel perspective on the robustness of lexical representations in L2 and further enriches the theoretical constructs of bilingual lexical processing models such as the BIA + and the Multilink Framework.

### The role of number of items per condition

A significant and positive influence of the number of items in each condition was shown on phonological priming effects. This aligns with a similar effect identified in the meta-analysis of L2-to-L1 translation priming studies (Wen & van Heuven, 2017). One possibility is that using more items reduces the noise in the data, thereby increasing the priming effect size (Van den Bussche et al., 2009; Wen & van Heuven, 2017). More items can also improve statistical power in priming studies (e.g., Dimitropoulou et al., 2011; Nakayama et al., 2016). While it is beyond the scope of this meta-analysis to provide a recommendation regarding the number of items per condition, the observed effect underscores the importance of using an adequate number of items when investigating phonological priming. Furthermore, it is recommended that a priori power analysis is conducted to determine the optimal number of items for a given study (for a tutorial on power analysis, see Brysbaert & Stevens, 2018). Brysbaert and Stevens (2018) also showed quite convincingly that the effect size will increase as the number of items increases because the noise of the estimates decreases.

### Limitations and future directions

The first limitation of this meta-analysis lies in the relatively modest sample size (*k*_*1*_ = 23, *k*_*2*_ = 75, *n* = 1387) and the unequal numbers of effects within each cell of different moderators. The limited sample size becomes especially pertinent within the context of a meta-regression analysis on an unbalanced data structure. This imbalance is primarily due to researchers’ preference for a specific task, priming direction, and script distance in empirical practice. For instance, there was a lack of cross-script studies employing the perceptual identification task, the word-naming task was used less frequently than the lexical decision task, and a very small number of within-script effects were identified (*k*_*2*_ = 7). Due to the limited number of within-script studies, we were not able to compare the effect sizes across within- and cross-script studies. Thus, further research is warranted to examine cross-language phonological priming utilizing less common designs, such as the perceptual identification task and the word-naming task. Additionally, there is a need to extend the investigation to the priming effect from L2 to L1, given the scarcity of studies dedicated to this priming direction. These future efforts will help minimize potential biases in understanding the intricate nature of phonological co-activation in bilingual lexicon.

Second, several studies (*k*_*1*_ = 4) were excluded due to insufficient data reported for effect calculation. This illuminates the need for promoting open-access data in the scientific community. There is also a pressing need for standardization in reporting results for future publications in academia, such as reporting both marginal means and standard errors for RTs and accuracy rates. A more standardized reporting schema would substantially facilitate transparency and reproducibility of scientific research, greatly benefiting future systematic and quantitative reviews. Third, we noticed that the majority (85%) of the included studies investigated English as the L2, possibly due to the prevalence of learning English as an L2 or foreign language. While our primary focus is not on a specific L2, it is crucial to acknowledge that our results may overly represent the phonological processing and activation of English learners. To mitigate this potential bias and promote a more comprehensive understanding of cross-language phonological priming, future studies should strive for a more balanced representation of L2 populations. By including a more diverse range of L2 populations, we can obtain a more nuanced and holistic understanding of cross-language phonological priming across different languages. Fourth, we acknowledged it as a limitation here that future meta-analyses should synthesize the data from mixed-effect models when they become more available in literature.

Lastly, as the current meta-analysis showed strong evidence for cross-language phonological activation, the next step is to delve deeper into the specifics of this process. For instance, future studies can investigate the temporal aspects of cross-language phonological activation, including the time course in terms of when it occurs and how it unfolds throughout visual word recognition. Understanding the temporal dynamics will shed light on the sequence of events involved in phonological activation and their underlying mechanisms. Another topic worthy of more research is whether cross-language phonological activation functions at a sublexical or lexical level. Pinpointing the locus of cross-language phonological activation will shed light on the level at which L1 and L2 phonological representations are integrated and co-activated. Previous studies have suggested that the phonological priming effect occurs at the sublexical level, based on the absence of influences from target word frequency and participants’ L2 proficiency. There have been empirical studies directly examining the effects of target word frequency and participants’ L2 proficiency on cross-language phonological priming. Future research needs to include frequency and proficiency as potential moderators in either meta-analysis or mega-analysis (synthesizing the raw data) to enhance our understanding of the origins of cross-language phonological activation.

## Conclusion

Our meta-analysis revealed significant phonological priming effects across different priming directions, task types and script distances. We also showed a significant effect of task type that the cross-script phonological priming is stronger in lexical decision compared to word naming, possibly due to the greater phonological demand in word naming and the greater need for inhibition in phonological activation in a dual-language setting. In addition, the magnitude of the phonological priming effect increases with a greater number of items in each condition, pointing to the important role of statistical power. More future research is called for on bilingual lexicon using different tasks, priming directions and languages with the same or different scripts, so that these potential moderators can be systematically examined and directly compared.

## Data Availability

The datasets generated during and/or analyzed during the current study are available via the Open Science Framework at osf.io/7fdbc/.

## Footnotes

1 In this meta-analysis, we examined the priming effect with RT as the primary outcome measure. However, for the six studies that only reported accuracy, we incorporated accuracy as an alternative outcome metric.

2 Since SOA and ISI were moderately correlated (*r* =.51, *p* <.0001), ISI was regressed on SOA, and the residual of ISI was entered into the regression model to reduce collinearity (see also Wen & Van Heuven 2017).

3 In this section, we reported the average effect sizes without excluding outliers. However, when examining moderator effects in the subsequent meta-regression analyses, several outlying and influential effects were excluded, resulting in different values of the effect sizes for the lexical decision and naming tasks (see details in the *Sensitivity analysis* section).

4 The cutoff value was calculated via the following formula: $\frac{2}{\sqrt{n}}$, where *n* is the number of included effects (Belsley et al., 1980, p. 28).

5 Note that effect #10 (Kim & Davis, 2003, Experiment 2) also had a large* ΔQ*_*E*_, however, the results of regression coefficients did not change after removing this effect.

## Code availability

All codes for the statistical analyses are available via the Open Science Framework at osf.io/7fdbc/.
